# Evaluation of the Antimalarial Potential of a Combination of *Terminalia macroptera* and *Erythrina sigmoidea:* In Vitro and In Silico Assays

**DOI:** 10.1155/japr/2472782

**Published:** 2026-05-03

**Authors:** Tientcheu Noutong Jemimah Sandra, Noumedem Anangmo Christelle Nadia, Yamssi Cedric, Gamago Nkadeu Guy-Armand, Ngouyamsa Nsapkain Aboubakar Sidiki, Mounvera Abdel Azizi, Vincent Khan Payne, Haibo Hu

**Affiliations:** ^1^ Department of Animal Biology, Faculty of Science, University of Dschang, Dschang, Cameroon, univ-dschang.org; ^2^ Laboratory of Tropical and Emerging Infectious Diseases, Dschang, Cameroon; ^3^ Department of Microbiology, Haematology and Immunology Faculty of Medicine and Pharmaceutical Sciences, University of Dschang, Dschang, Cameroon, univ-dschang.org; ^4^ Department of Biomedical Sciences, Faculty of Health Sciences, University of Bamenda, Bambili, Cameroon, unibda.net; ^5^ National Engineering Research Center for Modernization of Traditional Chinese Medicine–Hakka Medical Resources Branch, School of Pharmacy, Gannan Medical University, Ganzhou, China, gmu.cn

**Keywords:** ADME, antioxidant, cytotoxicity, *E. sigmoidea*, in silico, *Plasmodium falciparum*, *T. macroptera*

## Abstract

**Background:**

Due to the increasing resistance of malaria parasites to antimalarial drugs, medicinal plants are increasingly being employed to cure malaria. The purpose of this study is to establish the cytotoxicity, antioxidant, and antiplasmodial activity of a mixture of *Terminalia macroptera* and *Erythrina sigmoidea* extracts, two plants that have been traditionally used together in the management of malaria in Cameroon′s Western Region.

**Methods:**

The stem bark extracts of both plants were formulated according to established protocols. Previous studies reported the use of blends of *E. sigmoidea* and *T. macroptera* in antiplasmodial assays employing established methods. Three combinations were used: 75% *T. macroptera* and 25% *E. sigmoidea* in Combination 1, 50% *T. macroptera* and 50% *E. sigmoidea* in Combination 2, and 25% *T. macroptera* and 75% *E. sigmoidea* in Combination 3. The in vitro antiplasmodial activity of the combinations against chloroquine‐sensitive (3D7) and chloroquine‐resistant (Dd2) *Plasmodium falciparum* strains was evaluated by the SYBR green fluorescence‐based assay. Antioxidant activity was evaluated by DPPH (1,1‐diphenyl‐2picrylhydrazyl), FRAP free radical scavenging (ferric reducing antioxidant capacity), H2O2 (hydrogen peroxide), and NO (nitric oxide) radical scavenging. Cytotoxicity of the extracts was evaluated by using Vero cell line and human donor red blood cells (RBCs). Docking studies were conducted using the Schrodinger Maestro software.

**Result:**

Extracts of *E. sigmoidea* and *T. macroptera* were effective in all the three combinations. Being effective with IC_50_ values of 4.64 ± 0.09 *μ*g/mL for the 3D7 strain and 4.67 ± 0.24 *μ*g/mL for the Dd2 strain of *P*. *falciparum*, the third combination proved to be the most effective. The free radical scavenging activity of all the plant extracts was good. Against DPPH, FRAP, NO, and H_2_O_2_, IC_50_ values for the combination were 510.3 μg/mL, 159.6 μg/mL, and undetermined, respectively. Plant extract Combination 3 was not cytotoxic in cytotoxicity assay with CC_50_ of 217.05 ± 0.21 *μ*g/mL against Vero cells and reduced hemolysis compared with positive control. Phytochemical analysis of plant extract identified the existence of alkaloids, triterpenoids, tannins, quinones, saponins, flavonoids, and phenolics. Sigmoidin A, Bidwillon A, Abyssinone V, Flavogallonic acid, 3,4,5‐tri‐O‐caffeoylquinic acid were the Top 5 ligands that had the highest docking scores. Compounds are docked to *PfDHFR* because it is an essential enzyme, a validated antimalarial drug target, and docking provides mechanistic insight, resistance relevance, and predictive support for antiplasmodial activity.

**Conclusion:**

These findings suggest that the combination of stem bark extracts of *T. macroptera* and *E. sigmoidea* may have antimalarial activity. Additional in vivo antimalarial and toxicity studies are required for the scientific validation of the application of the two plant extracts in combination for antimalarial activity. The investigation of *T. macroptera* and *E. sigmoidea* via their interactions with proteins supported our hypothesis that the active substance is a strong inhibitor of the *Plasmodium falciparum* dihydrofolate reductase‐thymidylate synthase (*Pf*‐DHFR‐TS). Analyzing these compounds′ ADME characteristics contributes to the possibility of these ligands being developed into pharmaceutical forms.

## 1. Introduction

The most dangerous disease with regard to morbidity and mortality rates, especially in regions like Africa, Asia, and Latin America, is malaria [1]. This disease is primarily caused by a parasite which is called *Plasmodium* and is transferred through mosquito bites. There are five species of *Plasmodium* that people can contract and spread. *Plasmodium falciparum* is mostly responsible for severe illness. Parasites first proliferate in the liver, followed by erythrocytes. Generally, symptoms of malaria can be spotted around 10–15 days after the infected mosquito bite; these symptoms generally include fever, headaches, and vomiting. In case malaria is not treated on an urgent basis, it can become life threatening in no time.

Malaria remains a serious global health challenge, with an estimated 282 million cases and 610,000 deaths in 2024—roughly 9 million more cases than the previous year [2]. The WHO African Region continues to bear the greatest burden, with 11 countries accounting for about two‐thirds of global cases and deaths. Progress in reducing the malaria mortality rate nevertheless remains far off track.

According to WHO, RTS, S/AS01 vaccine is given to children from the age of 5 months in some African countries, but does not aim at complete prevention of the disease. This region continues to face severe health issues due to malaria. Pregnant women, and children below the age of five remain the most vulnerable group to this disease. [3]. Malaria is a serious concern in the public health sector of Cameroon, although surveillance systems and some indicators have shown improvement over time [4].

Malaria parasites have become resistant to several antimalarial medications in various parts of the world. The WHO′s [5] support of artemisinin‐based combination therapies (ACTs) as a malaria treatment policy may serve as a catalyst for further research into herbal combinations with documented ethnomedical antimalarial usage as a viable strategy for the identification of potential medications [6]. Because phytotherapy is often a combination therapy, it is thought to counteract the malarial *Plasmodium* parasites′ growing resistance to the conventional medications used to treat the disease, such as chloroquine. Similarly, the preferred course of treatment for malaria in conventional medicine is ACT [7]. In the humid tropics, folk medicine uses the stem bark of *Terminalia macroptera* and *Erythrina sigmoidea* to cure feverish diseases, including malaria [8]. Winstanley and Ward [9] suggested using combination therapy to circumvent medication resistance. Other research has suggested that to lessen medication resistance, combination treatments with quick clearance rates should be used. Compared with monotherapies, combination therapy offers a number of benefits [5].

Combination drug therapy is founded on the assumption that resistance is delayed if many targets with various modes of action are to be mutated. The key to an effective drug combination is that, even with resistance, there is still at least one drug with clinical activity. Additive, synergistic, or antagonistic combination therapy is all possible. The above plants, it is in Cameroonian traditional healers′ opinion, may be mixed together [10]. *E*. *sigmoidea,* Fabaceae family, commonly known as Mah quat, and *T. macroptera*, Combretaceae family, commonly known as Sareh, both enjoy wide use in Cameroonian traditional medicine [11]. Ethnomedicinal reports indicate that different plant parts, including leaves, roots, seeds, and stem bark, are used by traditional healers for the management of fevers, hepatitis, venereal diseases, conjunctivitis, piles, diarrhea, dysentery, malaria, microbial infections, jaundice, and syphilis [12].

Most sub‐Saharan African nations, including Cameroon, have medicinal plants like *T. macroptera* and *E. sigmoidea*. The people of Noun Division, West Region, Cameroon, use the stem bark, leaves, and roots of these plants to treat malaria. Sidiki et al. [11, 13] have proved the antimalarial property of *T. macroptera*, and Jemimah et al. [14] have proved the antimalarial property of *E. sigmoidea*. Most parasites, can induce a cascade of oxidative reactions in the host. The parasite′s digestive vacuole breaks down hemoglobin into amino acids, releasing toxic free heme and generating an overload of radical species, the primary perpetrator of oxidative stress [15]. A more effective mechanism for preventing malaria that would be less damaging to the host would be to take an antioxidant supplement. The traditional therapists of the Western Region of Cameroon cure malaria with a combination of *T. macroptera* and *E. sigmoidea*, which is shown by this research to possess antiplasmodial and antioxidant activity.

## 2. Material and Methods

### 2.1. Plant Selection

Two species were selected for this study based on ethnobotanical research conducted in Noun city with the collaboration of traditional medicine practitioners. The plants were harvested from a forest (Ndjindare) in Noun Division, which is located at the border between Adamawa and West Regions of Cameroon, in Foumban, in January 2023 during the dry season. In January 2023, an ethnobotanical investigation was undertaken to gather information on medicinal plant species, symptoms managed, and the preparation and administration of herbal treatments. From the interviewed herbalists, a total of 12 plant species were mentioned. There was, however, general agreement among the herbalists on the use of four of these: *T*. *macroptera, Khaya grandifoliola, E*. *sigmoidea,* and *Lophira lanceolata*. *T. macroptera* and *E*. *sigmoidea* are both abundant in Cameroon and have previously documented antiplasmodial properties. These two plants are traditionally used in combination for the treatment of malaria. Therefore, it was essential to first evaluate their individual antiplasmodial activities before assessing the activity of their combined extracts.

### 2.2. Chemicals and Reagents Agents

To measure the activity of the plant extracts, antiplasmodial assays used chloroquine, a broad antimalarial agent, as the positive control. To ensure physiological pH and osmolarity in in vitro assays, samples were diluted and prepared using distilled water and phosphate‐buffered saline (PBS). Extracts used in the bioassays were dissolved in 1% dimethyl sulfoxide (DMSO), which served as a nontoxic solvent. During the calculation of parasitemia, the use of Giemsa stain was a necessary step when identifying *Plasmodium* species on the microscopic examination. Other materials and reagents that were to be applied or utilized had every caution chosen as pure along with a view toward observing adherence to routine lab techniques.

### 2.3. Plant Material

The plant materials, such as stem bark, leaves, seeds, and flowers, were taken to the National Herbarium of Cameroon in Yaounde for identification. The plant was authenticated by Dr. Ngantsop, a taxonomist from the Department of Botany, University of Yaounde, Cameroon. For the voucher herbarium specimens, the Reference Codes 3053/SRFK for *T. macroptera* and 8749/SRFCAM for *E. sigmoidea* were recorded.

### 2.4. Preparation of Extracts

The report indicated that it is traditional practitioners who prepare this medicine, by macerating palm wine, which is why extraction was performed using ethanol and previous studies on these plants have shown that the ethanol extract has the higher activity. The ethanol extract was made according to the method written by Wabo et al. In brief, two 3‐L flasks were filled with 100 g of the powder, which weighed on an SF‐400 electric balance. The two flasks were then charged 1 L of 95% ethanol per flask, shaken for 5 min. The mixed solutions were then macerated for 72 h at room temperature. They were first filtered through a 150‐*μ*m sieve before they went through Wattman filter paper (N°1). The filtrates so achieved were dried in an oven at 40°C to yield the dry ethanol extract [16]. The resulting ethanol extracts were kept under refrigeration at 4°C until required for subsequent analysis.

#### 2.4.1. Extraction Yield

Percentage yields were calculated for both species using the following formula.

Percentage yield=weight of dried extract weight of dried plant sample×100

Following ethanol extraction of 100 g of bark powder, yields of 6.56% and 8.1% were recorded for *T. macroptera* and *E. sigmoidea*, respectively.

### 2.5. HPLC‐PDA‐MS Analysis

UHPLC‐PDA‐ESI‐MS analysis was performed using a Shimadzu LC‐2030C ultrahigh‐performance liquid chromatography system (Shimadzu Corporation, Kyoto, Japan) coupled to a UV/Vis diode array detector (DAD) and a single‐quadrupole LCMS‐2020 mass spectrometer (Shimadzu Corporation, Kyoto, Japan). Instrument control, data acquisition, and processing were carried out using LabSolutions software (Shimadzu Corporation, Kyoto, Japan). Chromatographic separation of metabolites was achieved on a Poroshell 120 EC‐C18 column (100 × 3.0 mm, 2.7 *μ*m; Agilent Technologies, Santa Clara, California, United States), maintained at a column temperature of 35°C using the integrated column oven of the LC system (Shimadzu Corporation, Kyoto, Japan).

The mobile phase consisted of ultrapure water containing 0.1% (*v*/*v*) formic acid (Sigma‐Aldrich, St. Louis, Missouri, United States) as Mobile Phase 1 (MP1) and HPLC‐grade acetonitrile containing 0.1% (*v*/*v*) formic acid (Sigma‐Aldrich, St. Louis, Missouri, United States) as Mobile Phase 2 (MP2). Ultrapure water was obtained using a Milli‐Q water purification system (Millipore, Bedford, Massachusetts, United States). The flow rate was set at 0.40 mL/min, and the injection volume was 2.0 *μ*L using the autosampler of the LC‐2030C system (Shimadzu Corporation, Kyoto, Japan).

The gradient elution program (MP1:MP2) was as follows: 0.00–0.50 min, 95:5 (isocratic); 0.50–8.00 min, linear gradient to 60:40; 8.00–12.00 min, linear gradient to 5:95; 12.00–13.00 min, held at 5:95 for column washing; 13.00–13.50 min, linear return to 95:5; and 13.50–16.00 min, re‐equilibration at 95:5. PDA detection was conducted over a wavelength range of 200–600 nm with a scan rate of 0.5 s per scan using the integrated DAD (Shimadzu Corporation, Kyoto, Japan).

Mass spectrometric detection was performed using an electrospray ionization (ESI) source operating in both positive and negative ionization modes on a single‐quadrupole mass spectrometer (LCMS‐2020; Shimadzu Corporation, Kyoto, Japan). The optimized ESI parameters were as follows: capillary voltage of 3.5 kV, fragmentor voltage ranging from 80 to 120 V (optimized per analyte), fixed collision‐induced dissociation (CID) energy of approximately 20 eV, and a mass scan range of m/z 100–1000. High‐purity nitrogen was used as both the nebulizing and drying gas (Air Liquide, Douala, Cameroon) at a flow rate of approximately 10 L/min, with a nebulizer pressure of about 40 psi and an interface temperature set at 300°C.

### 2.6. Parasite Culture and Maintenance

With a modified Trager and Jensen method, the parasites′ asexual erythrocytic stages were cultured continuously with consistent growth and reproducible experimentation. Both chloroquine‐resistant (Dd2) and chloroquine‐sensitive (3D7) strains from the American Type Culture Collection (ATCC) constituted a powerful model on which to base drug efficacy comparisons. Using both strains enabled the examination of mechanisms of resistance, making the study more directly applicable to antimalarial drug design and offering greater translational potential for clinical use [17].

### 2.7. Antiplasmodial Activity In Vitro

The antiplasmodial activity was carried out according to the method described by Trager and Jensen [18] with slight modifications. Briefly, the 3D7 chloroquine‐sensitive strain of *P*. falciparum and the multidrug‐resistant strain Dd2 of *P*. *falciparum* were cultured in 500‐mL RPMI 1640 (Gibco, United Kingdom) with 25‐mM HEPES (Gibco, United Kingdom), 0. 50% (*w*/*v*) Albumax I (Gibco, United States), 1× hypoxanthine (Gibco, United States), and 20‐*μ*g/mL gentamicin (Gibco, China) with fresh human group O Rhesus–positive red blood cells at 4% hematocrit. The cells were then incubated in a gas humidified incubator with 92% N_2_, 5% CO_2_, and 3% O_2_ at 37°C. Fresh RPMI media were added to the medium every day to enhance parasitic growth in culture [18].

Plant extracts combinations and reference drugs were prepared as stock solutions in DMSO and further diluted with complete RPMI 1640 medium to obtain the desired test concentrations. The final concentration of DMSO in all assay wells did not exceed 0.5% (*v*/*v*), a level shown not to affect parasite growth. Antiplasmodial activity was assessed in 96‐well microplates. Negative control wells contained infected erythrocytes with culture medium and 0.5% DMSO, whereas positive control wells contained infected erythrocytes treated with a standard antimalarial drug (chloroquine or artesunate). Untreated infected cultures served as the growth control, and uninfected erythrocytes were included to monitor background effects.

Parasitemia and parasite developmental stages were assessed after incubation by preparing thin blood smears, which were stained with Giemsa and examined microscopically under a 100× oil‐immersion objective. Percentage inhibition of parasite growth was calculated relative to the negative control, and IC_50_ values were determined from dose–response curves. All experiments were performed in triplicate.

#### 2.7.1. Culture Synchronization

Parasite synchronization was done according to the method described by Lambros and Vanderberg [19]. Briefly, parasite cultures having over 80% of ring stages were synchronized to the same stage (ring stage) by preincubation in sorbitol 5% (*w*/*v*) during 10 min. Using synchronized cultures at the same developmental stage instead of mixed‐stage cultures, we were able to ascertain the activity of *T. macroptera* and *E. sigmoidea* combined extracts on the three stages of the life cycle (rings, trophozoite, and schizont) of *P. falciparum* [19].

#### 2.7.2. Preparation of Test Combinations

To make 100 mg/mL concentrated stock solutions of the extracts, 100 mg of all samples were dissolved in 1 mL of DMSO. The stock solutions were then mixed in the following proportions as indicated in Table 1 below:

**Table 1:** Combinations of extracts.

Combinations	*E. sigmoidea*	*T. macroptera*
C_1_	25 *μ*L	75 *μ*L
C_2_	50 *μ*L	50 *μ*L
C_3_	75 *μ*L	25 *μ*L

#### 2.7.3. Preparation of Intermediate Concentrations of Combination and Positive Control (Artemisinin and Chloroquine)

For the preparation of intermediate concentrations of each sample (combinations), 198 *μ*L of incomplete RPMI1640 medium was mixed with 20 *μ*L of artemisinin (100 *μ*M) and 180 *μ*L of chloroquine (100 *μ*M) stock solutions, respectively, and 2 *μ*L of extract stock solution (100 mg/mL) were transferred to the 96‐well microplate. Geometric dilution of order five ensued. Artemisinin and chloroquine intermediate concentrations ranged from 0.016 to 10 *μ*M, and crude extract concentrations from 1.6 to 1000 *μ*g/mL.

#### 2.7.4. SYBR Green Fluorescence‐Based *P. falciparum* Growth Inhibition Test (IT) In Vitro

Against cell growth, chloroquine, artemisinin, and plant extracts were tested in 96‐well flat‐bottom microplates with SYBR Green I fluorescence to assess antiplasmodial activity. SYBR Green I′s sensitivity to generate intensive fluorescence on contact with DNA is the reason why it can be used to estimate cell growth. Since human red blood cells where the parasite lives are nonnucleated, SYBR Green I will be used only to track the growth of the plasmodia. During the experiment, 90‐*μ*L ring‐synchronized parasite suspension 2% parasitemia, 1% hematocrit was inoculated with 10‐*μ*L different volumes of prediluted extract, artemisinin, and chloroquine combinations. The plates were incubated in a humidified incubator with 92% N_2_, 5% CO_2_, and 3% O_2_ for 72 h at 37°C. Artemisinin and chloroquine concentrations in the final 100‐*μ*L volume ranged from 0.0016 to 1 *μ*M (DMSO, 0.1%), and the plant extract concentrations in the test plates ranged from 0.8 to 100 *μ*g/mL (DMSO < 1*%*). Experiments were performed twice [20].

One hundred microliters of SYBR Green I buffer [6 *μ*L of 10,000 × SYBR Green I (Invitrogen) + 600 *μ*L of red cell lysis buffer {TRIS (25 mM; pH 7.5)} + 360 *μ*L of EDTA (7.5 mM) + 19.2 − *μ*L parasite lysis solution {saponin (0.012%; wt/vol)} and 28.8 *μ*L of Triton X‐100 (0.08%; vol/vol)}] were carefully added to each well after a 72‐h incubation period. After that, incubation was carried out at 37°C for an hour in the dark. After an hour of incubation, the fluorescence was measured using the Infinite M200 plate reader (Tecan). The wavelengths of excitation and emission were 485 and 538 nm, respectively. All experiments were performed in triplicate to ensure accuracy and reproducibility.

### 2.8. Cytotoxicity Test on Vero Cells

The cytotoxic effect of the combinations was also evaluated using the resazurin‐based assay described by Bowling et al. [21] on Vero cells. Briefly, Vero cells were grown well in Dulbecco′s Modified Eagle medium (DMEM), made up of 13.5‐g/L DMEM (Sigma Aldrich), 10% fetal bovine serum (Sigma Aldrich), 0.2% (*w*/*v*) sodium bicarbonate (Sigma Aldrich), and 50 *μ*g/mL gentamicin (Sigma Aldrich). Vero cells were seeded at a seeding density of 1 × 10^4^ cells/well into 96‐well flat‐bottomed cell culture plates in 100 *μ*L of complete medium. To facilitate cell adhesion, they were incubated for 24 h at 37°C with 5% CO_2_. After adhesion, test plates were seeded with 10 *μ*L of each series‐diluted test sample solution (combinations), and the plates were incubated for 48 h under the same experimental culture conditions [21].

The experiment plates had a growth control well (0.1% DMSO—100% growth) and a positive control well (20‐*μ*M podophyllotoxin) after medium replacement with full media. Cell growth was quantified at the end of incubation after adding 10 *μ*L of a stock solution of resazurin (0.15 mg/mL in sterile PBS) to each well. Incubation of wells for 4 h under the same culture conditions followed. Afterwards, the 530/590‐nm excitation/emission fluorescence was read by an Infinite M200 brand Tecan fluorescence multiwell plate reader. 50% cytotoxic concentrations (IC_50_) were used to report the data. All experiments were performed in triplicate to ensure accuracy and reproducibility.

### 2.9. Hemolysis or Cytotoxicity Test on Red Blood Cells

The hemolysis test against healthy erythrocytes was carried out according to the method described by Sinha et al. [22]. The test was therefore established to make sure that the antimalarial activity obtained with the combinations would not be explained by host lysis of the parasite, erythrocytes. Experimentally, 500 *μ*L of 4% hematocrit suspension of fresh group O^+^ blood normal erythrocytes (obtained from the blood bank of the district hospital of Dschang) were incubated in incomplete RPMI 1640 in Eppendorf tubes with 500 *μ*L of the mixtures at different concentrations. Erythrocyte suspension in complete culture media at 4% hematocrit and Triton X‐100 to 0.5% (for 100% hemolysis), respectively, were used to create the positive and negative controls under the same conditions.

Test plate working concentrations (Triton X‐100 0.5% and the 0.5% DMSO combinations) were between 1000 and 62.5 *μ*g/mL for a final volume of 1000 *μ*L. Plates were incubated for 3 h at 37°C with 5% CO_2_. After incubation and centrifugation for 2500 rpm for 3 min, the supernatant absorbance of hemoglobin release was measured at 540 nm with Infinite M200 microplate reader (Tecan). All experiments were performed in triplicate to ensure accuracy and reproducibility. After 3‐h incubation, the hemolysis rate of every combination was measured using the following formula as a percentage of total hemolysis (positive control):

%hemolysis=absorbance of sample−absorbance of blank sample absorbance of positive control×100

Interpretation:

• Samples causing ≤ 5% hemolysis were considered nonhemolytic.
• The effective concentration causing 50% hemolysis (EC_50_) was determined from dose–response curves. When EC_50_ exceeded the highest tested concentration (1000 *μ*g/mL), it was reported as > 1000 *μ*g/mL.

All experiments were performed in triplicate to ensure reproducibility.

### 2.10. The Most Active Combination of In Vitro Antioxidant Activity

We continue our experiment with the Combination 3 (C_3_) because this combination presents a higher activity than the two other ones.

#### 2.10.1. DPPH Antiradical Activity

The DPPH radical scavenging activity was carried out according to the method described by Popovici et al., [23]. Vitamin C or plant extract combination (in varying final concentrations: 1, 3, 10, 30, 100, and 300 *μ*g/mL) was combined in 24 *μ*L each with 153 *μ*L of methanol in spectrophotometer cuvettes, and absorbance at 540 nm was taken as initial. Then, 80 *μ*L of DPPH (0.063 mg/mL) was added, and the reaction was kept in darkness for 20 min. The blank consisted of 153 *μ*L of methanol and 80 *μ*L of DPPH. The absorbance was recorded after the addition of the solution of DPPH to the reaction medium. There were six replicates taken for testing, and vitamin C was used as a positive control.

A=AO−A1AO×100

A0: DPPH absorbance, A1: sample absorbance,IC_50_ : concentration of the sample necessary to neutralize 50% of the free radicals) was obtained.

#### 2.10.2. Ferric Reducing Power

The reducing power of *T. macroptera* was performed accordingly as previously described by Bokhari et al. [24]. Test tubes held the addition of vitamin C or plant combination in the quantity of 200 *μ*L, phosphate‐buffered solution in a quantity of 0.5 mL (200 mM, pH = 6.2), and potassium ferricyanide in a quantity of 0.5 mL. After this, the test tubes were incubated for 20 min at 50°C. The samples were centrifuged at 10 min at 3000 rpm since 0.5 mL of tricholoroacetic acid was added during incubation. A total of 0.1 mL of FeIII was added in each tube after 0.5 mL of the supernatant and 0.5 mL of water were mixed. A total of 700‐nm absorbance was measured following incubation with the mixture for 10 min at 37°C. Final plant extract and vitamin C concentrations were 1, 3, 10, 30, 100, and 300 g/mL (30 mM). Six replicates of the test were performed.

#### 2.10.3. Nitric Oxide (NO) IT

The method described by Sidiki et al. [25] was used to evaluate the NO inhibition. The nitrite ions produced by combining oxygen and NO in vitro at physiological pH can be found using the NO IT Griess reaction. Thus, in test tubes, 180 *μ*L of extract or vitamin C (1, 3, 10, 30, 100, or 300 g/mL) was combined with 1520 *μ*L of nitroprusside (10 mM) and incubated for 2.5 h at 25°C. The solution was incubated for 5 min in darkness after the removal of 500 *μ*L of it and the addition of 500 *μ*L of 1% sulfanilamide. Five minutes were then allowed to pass in darkness after the addition of 500 *μ*L of 0.1% nathylethylenediamine (NED). The reading of absorbance at 530 nm was taken with the microplate reader mentioned above [26]. Six replicates of the test were performed. The formula above was employed to compute scavenging activity:

A=absorbance control−absorbance sampleabsorbance control

#### 2.10.4. Activity of Hydrogen Peroxide Scavenging

The method described by Ruch et al. [27] was used to find out the capability of the plants combination toward the degradation of hydrogen peroxide. In this case, 0.6 mL of solution of hydrogen peroxide (40 nM) was mixed with phosphate buffer solution (pH = 7.4) and incubated for 50 min. After the addition of 0.4 mL of plant extract with varying concentrations (1, 3, 10, 30, 100, or 300 g/mL), the sample was blended at room temperature and left at room temperature for 10 min. Six replicates of the test were performed. The absorbance at 230 nm was read by a spectrophotometer [27].

%H20=absorbance control−absorbance sampleabsorbance control

### 2.11. Docking Simulations

Ligand preparation was performed using the LigPrep module of Schrödinger Maestro (Version 12.5). All ligands were converted into three‐dimensional structures, and possible ionization states and tautomers were generated at physiological pH (7.0 ± 2.0) using the Epik tool. Chiral centers were retained as specified, and geometry optimization was carried out using the OPLS3e force field. Low‐energy conformers were generated prior to docking.

Molecular docking was carried out using the Glide module in both standard precision (SP) and extra precision (XP) modes. Initial screening of compounds was performed using SP docking, followed by XP docking of the top‐ranked ligands to refine binding poses and improve scoring accuracy. For each ligand, a maximum of 10 docking poses was retained, and the best ranked pose was selected based on GlideScore and Emodel values.

The receptor grid was centered on the cocrystallized ligand binding site for both the wild‐type *Plamodium falciparum* dihydrofolate reductase‐thymidylate synthase (*Pf*‐DHFR‐TS) (PDB ID: 1J3I) and mutant *Pf*‐DHFR‐TS (PDB ID: 8JFC) structures. Default van der Waals scaling factors (0.80 for receptor and 0.15 partial charge cutoff) were applied to account for receptor flexibility. No constraints were applied during docking to allow unbiased ligand binding

Docking results were evaluated using GlideScore, which estimates ligand–receptor binding affinity, and Emodel, which combines GlideScore, coulombic energy, van der Waals energy, and ligand strain to prioritize optimal binding poses. A comprehensive summary of GlideScore and Emodel values for all docked compounds in both wild‐type and mutant *Pf*‐DHFR‐TS systems is provided.

To validate the docking protocol, a redocking procedure was performed using the cocrystallized ligand of both wild‐type and mutant *Pf*‐DHFR‐TS (PDB IDs: [insert WT ID], 8JFC). The native ligand was extracted from each crystal structure and redocked into its respective binding site using the same Glide docking parameters applied to the test compounds. The accuracy of the docking protocol was assessed by calculating the root mean square deviation (RMSD) between the heavy atoms of the redocked ligand and the crystallographic conformation using the Maestro structural alignment tool. RMSD values of less than 2.0 Å were obtained for both wild‐type and mutant *Pf*‐DHFR‐TS, indicating good agreement between predicted and experimental binding poses and supporting the reliability of the docking methodology [28].

The grid box for the *Pf*‐DHFR‐TS chain was constructed partly by the Maestro′s Glide program during the Receptor Grid Generation step. By applying the Maestro Sitemap program, the active region position is expected. Two boxes of dimensions 15 × 15 × 15 and 20 × 20 × 20 (X:28.59, Y:5.84, Z:59.59) are constructed in order to find the grid center. The grid center was identified using these boxes that are a feature of the *Pf*‐DHFR‐TS active site. The Glide module of the Schrödinger Maestro software was used to perform docking studies [28].

Tables 2,3 and 4 show a summary results of the docking process. Molecular docking was employed to produce potential adduct structures, and they were ranked and classified according to the scoring ability of the software (Table 5) [29]. The three‐dimensional shape of any complex can be inferred based on the binding affinity of the target and ligand. The prediction of the orientation and conformation of a ligand in a given binding site is referred to as docking. It is “docking” and is the process. In the first step, the protein structure was also prepped by using Maestro′s “protein preparation wizard” Figure 1. In the next step, through modular automated state generation and optimization protocols, hydrogen atoms and necessary bonds were incorporated into the missing place in the protein molecule. After the optimization step, receptor grids were generated, and multiple docked ligand conformations were employed to study the docking scores [30–32].

**Table 2:** Information of main peaks of mixture of two plants extract.

No	*T*_R_ (min)	*m*/*z*(*M* − *H*)^−^	*m*/*z*(*M* + *H*)^+^	Formula	Annotation	Area (%)
1	1.20	389.159	387.145	C_21_H_24_O_7_	Orientanol A	TIC (ESI−) = 26.45, single XIC = 76.50, TIC (ESI+) = 24.77, TAC = 2.89, 254 nm = 1.42
2	1.33	482.965	480.95	—	—	TIC (ESI−) = 26.45, single XIC = 71.70, TIC (ESI+) = 24.77, single XIC = 21.07
3	3.66	455.025	453.01	C_21_H_10_O_12_	Flavogallonic acid	TAC = 7.28, 254 nm = 0.10
4	4.74	468.115	466.1	—	—	Single XIC = 100.00, TAC = 7.92, 254 nm = 10.44
5	4.83	468.065	466.05	—	—	Single XIC = 95.30, TAC = 7.92, 254 nm = 10.44
6	5.10	688.224	686.21	—	—	TIC (ESI−) = 5.17, single XIC = 49.11, TAC = 8.64, 254 nm = 12.85
7	5.20	392.972	390.958	—	—	TIC (ESI−) = 5.17, single XIC = 33.31, TAC = 0.02, 254 nm = 0.06
8	5.36	471.923	469.908	—	—	TIC (ESI−) = 5.17, single XIC = 72.25, TAC = 8.64, 254 nm = 12.85
9	5.74	593.82	591.805	—	—	TIC (ESI−) = 5.17, TAC = 8.96, 254 nm = 14.69
10	5.85	635.088	633.073	C_27_H_22_O_18_	Corilagin	TIC (ESI−) = 5.17, single XIC = 58.99, single XIC = 40.54, TAC = 0.04, 254 nm = 0.05
11	6.34	425.196	423.181	C_25_H_28_O_6_	Sigmoidin a	TIC (ESI−) = 9.69, single XIC = 6.19, single XIC = 12.21, TAC = 8.03, 254 nm = 15.04
12	6.36	423.18	421.166	C_25_H_26_O_6_	Sigmoidin f	TIC (ESI−) = 9.69, single XIC = 88.80, single XIC = 87.43, TAC = 8.03, 254 nm = 15.04
13	6.66	543.165	541.15	C_25_H_24_O_7_	Neocyclomorusin	TIC (ESI−) = 9.69, single XIC = 29.80, TAC = 6.28, 254 nm = 11.14
14	6.67	571.087	569.073	C_30_H_18_O_12_	—	TIC (ESI−) = 9.69, TAC = 6.28, 254 nm = 11.14
15	6.80	545.456	543.442	C_35_H_60_O_4_	*β*‐D‐glucopyranoside	TIC (ESI−) = 9.69, TAC = 6.28, 254 nm = 11.14
16	6.88	566.021	564.007	—	—	TIC (ESI−) = 9.69, single XIC = 81.82, TAC = 1.20, 254 nm = 1.41
17	7.04	534.015	532	—	—	TIC (ESI−) = 9.69, single XIC = 100.00, TAC = 1.86, 254 nm = 2.37
18	7.24	434.915	432.9	—	—	TIC (ESI−) = 9.69, single XIC = 79.61, TAC = 2.03, 254 nm = 3.65
19	7.28	449.015	447	—	—	TIC (ESI−) = 9.69, single XIC = 83.11, single XIC = 18.44, TAC = 2.03, 254 nm = 3.65
20	7.31	577.165	575.15	—	—	TIC (ESI−) = 9.69, single XIC = 59.96, single XIC = 84.05, TAC = 2.03, 254 nm = 3.65
21	7.38	448.965	446.95	—	—	TIC (ESI−) = 9.69, single XIC = 83.92, single XIC = 18.44, TAC = 2.03, 254 nm = 3.65
22	7.47	559.115	557.1	—	—	TIC (ESI−) = 9.69, single XIC = 84.16, TAC = 4.80, 254 nm = 8.81
23	7.57	513.15	511.135	—	—	TIC (ESI−) = 9.69, single XIC = 94.39, TAC = 4.80, 254 nm = 8.81
24	8.61	603.004	600.99	C_28_H_10_O_16_	Terminalin	TIC (ESI−) = 5.90, single XIC = 14.41, TIC (ESI+) = 10.22, TAC = 2.36, 254 nm = 2.42
25	8.64	603.004	600.99	C_28_H_10_O_16_	Terminolic acid	TIC (ESI−) = 5.90, single XIC = 14.41, TIC (ESI+) = 10.22, TAC = 2.36, 254 nm = 2.42
26	11.02	679.166	677.151	C_34_H_30_O_15_	3,4,5‐Tri‐o‐caffeoylquinic acid	TAC = 0.84, 254 nm = 1.15
27	11.91	551.265	549.25	—	—	Single XIC = 20.28, TAC = 10.94, 254 nm = 1.55
28	12.68	403.065	401.05	—	—	Single XIC = 24.60, TAC = 1.04, 254 nm = 0.24
29	15.09	423.217	421.202	C_26_H_30_O_5_	4‐Methylabyssinone V	TIC (ESI−) = 8.28, single XIC = 4.32, single XIC = 6.49, TAC = 0.24, 254 nm = 0.06
30	15.69	409.201	407.186	C_25_H_28_O_5_	Abyssinone V	TIC (ESI−) = 8.28, single XIC = 85.58, single XIC = 82.03, TAC = 0.62, 254 nm = 0.10
31	15.70	409.201	407.186	C_25_H_28_O_5_	Bidwillon A	TIC (ESI−) = 8.28, single XIC = 85.58, single XIC = 82.03, TAC = 0.62, 254 nm = 0.10

**Table 3:** Antiplasmodial activity of the crude extracts and combinations of *Terminalia macroptera* and *Erythrina sigmoidea* on the chloroquine‐sensitive (3D7) strain and chloroquine‐resistant (Dd2) strain of *P. falciparum.*

	Mean *I* *C*_50_ combination (*μ*g/mL)					Mean *I* *C*_50_	
	*Terminalia macroptera* Crude extract	*Erythrina sigmoidea* Crude extract	Combination 1	Combination 2	Combination 3	Artemisinin (*μ*M)	Chloroquine (*μ*M)
Chloroquine‐sensitive strain (3D7) of *P. falciparum*	5.46 ± 0.48	6.44 ± 0.08	4.93 ± 0.34^a^	5.02 ± 0.21^a^	4.64 ± 0.09^a^	0.02 ± 0.00	0.03 ± 0.00
Chloroquine‐resistant strain (Dd2) of *P. falciparum*	6.15 ± 1.46	7.53 ± 0.22	4.94 ± 0.34^a^	5.23 ± 0.14^a^	4.67 ± 0.24^a^	0.04 ± 0.01	0.64 ± 0.08

^a^Values sharing the same letter within a column are not significantly different at the 5% level.

**Table 4:** Cytotoxicity on Vero cells.

Combinations	*M* *e* *a* *n* *C* *C*_50_ ± *S* *D*	Selectivity index (SI)
C_1_	196.65 ± 1.3^a^	39.81
C_2_	202.8 ± 0.3^b^	38.78
C_3_	217.05 ± 0.2^c^	46.48
Podophyllotoxin (*μ*M)	0.53 ± 0.0	NA
DMSO (1%)	2.13 ± 0.1	NA

*Note:* Superscripted letters (a,b, and c) indicate that values sharing the same letter are not significantly different at the 5% level.

Abbreviation: NA, none applicable.

**Table 5:** Antioxidant activities of the combinations of extracts of *E. sigmoidea* and *T. macroptera.*

Tests	*I* *C*_50_(*μ*g/mL)	
	Combination 3	Vit C
DPPH	510.30 ± 120.38	4.77 ± 91.12
FRAP	Undetermined	159.60 ± 2.66
NO	Undetermined	54.22 ± 62.80
H_2_O_2_	1112 ± 9.44	Undetermined

*Note:* Undetermined means that the value is too high. Six replicates of the test were performed.

Figure 1(a) A Ramachandran plot of the *P. falciparum* dihydrofolate reductase‐thymidylate synthase (*Pf*‐DHFR‐TS) (PDB id: 1J3I), and (b) an optimized version of the *Pf*‐DHFR‐TS (surface mode) [33].

**Figure:**
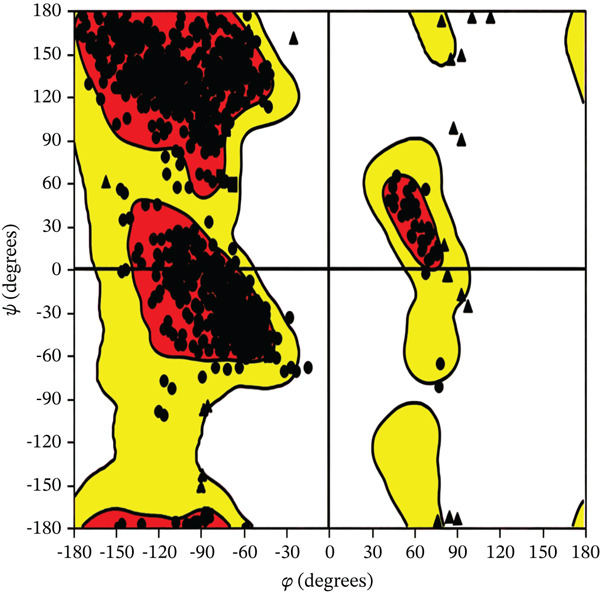
(a)

**Figure:**
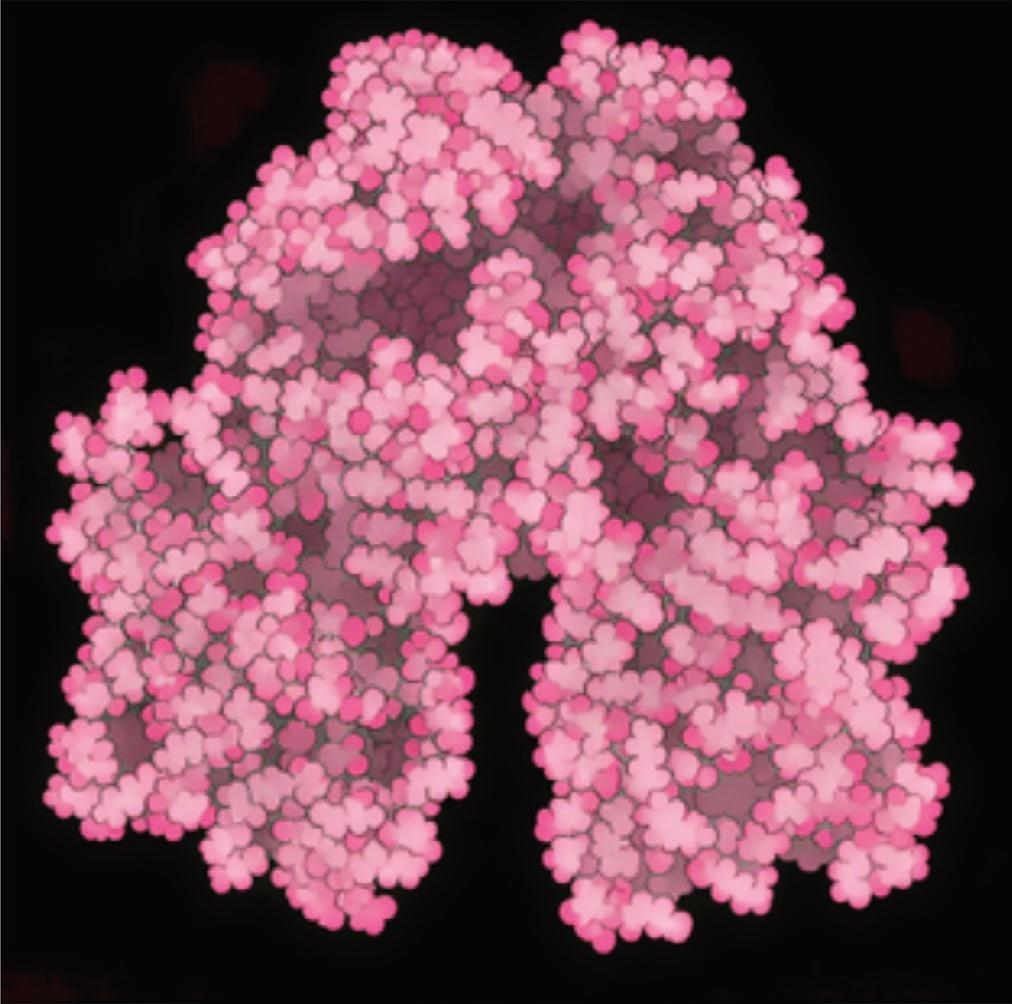
(b)

### 2.12. Evaluation of In Silico ADME

With the Qikprop application, the ADME properties of *T. macroptera* with *E. sigmoidea* stem bark chemicals were predicted [28] the Schrodinger suites are included in the Maestro program. These are the parameters that are considered to be standard for this rule:

1. The mass of the molecules should not be greater than 500.
2. A donor that is capable of forming a hydrogen bond (with a threshold of ≤ 5).
3. The range of hydrogen bond acceptors that could be considered acceptable is 10 or less.
4. LogP is extremely lipophilic with a value in the range of acceptability being ≤ 5.
5. Molar refractivity has a range of acceptability between 40 and 130.

The Schrödinger Maestro QikProp module (Version 12.5) was used to compute the ADME properties of the components [34].

### 2.13. Ethical Consideration

All authors hereby declare that the “Principles for the Care of Laboratory Animals” (NIH Publication No. 85‐23, revised 1985) have been followed, as well as specific national laws, where applicable. All experiments were reviewed and approved by the Institutional Ethics committee for research on Human Health Review Board (N°4846/CEI‐UDo/04/2023/T) of the University of Douala.

### 2.14. Statistical Analysis

The percentage inhibition was calculated using the Microsoft Excel software and the fluorescence data collected. The IC_50_ values were calculated by using the concentration–inhibition curves obtained by plotting the logarithm of concentration versus percent inhibition using the Graphpad Prism 8 software. Docking studies were conducted by the Glide module of Schrödinger Maestro software [28]. A number of potential structures for adducts formed through molecular docking were listed and ranked according to the score function by the software. ADME properties of compounds were calculated using the Schrödinger Maestro 12.5 QikProp tool [34].

## 3. Results

### 3.1. HPLC‐MS Analysis

Figure 2 shows UHPLC‐DAD‐ESI TIC− and TIC+ chromatograms of (A) the combined plant extract, (B) *E*. *sigmoidea,* and (C) *T*. *macroptera*. The combined extract exhibits a greater number of chromatographic peaks, indicating enhanced chemical complexity and the presence of diverse metabolites derived from both plant species. With the assistance of positive and negative ion mode HPLC‐MS, metabolite profiles of pairings of *T. macroptera* and *E. sigmoidea* stem bark ethanol extracts were obtained. With LC‐MS using patterns of two combinations of extract and single plant extract, qualitative analysis made putative annotation of 32 major peaks easy (Figure 2). These compounds are mostly found in both plants. Information about the annotation of the plants is presented in Table 2. However, there are still some compounds whose mass was detected but, due to lack of complete information, cannot be identified now.

Figure 2UHPLC‐DAD‐ESI, TIC^−^, and TIC^+^ chromatograms of (A) mixture of the plants, (B) *Erythrina sigmoidea,* and (C) *Terminalia macroptera.*

**Figure:**
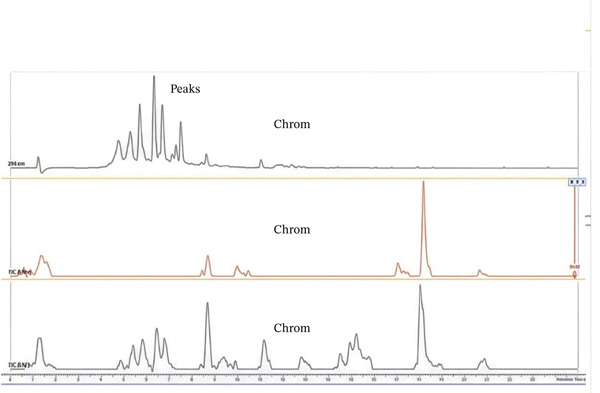
(A)

**Figure:**
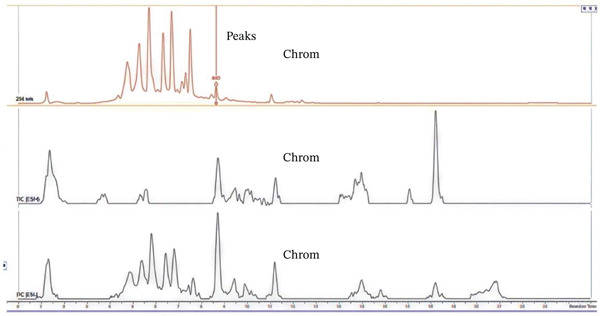
(B)

**Figure:**
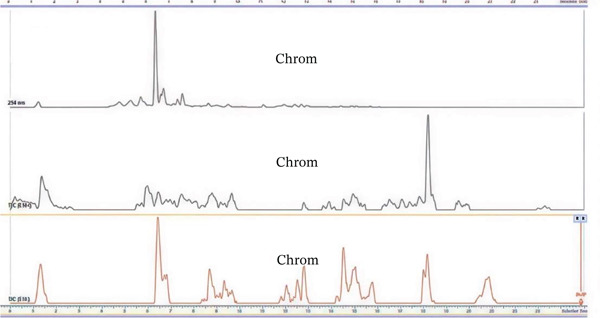
(C)

### 3.2. In Vitro Antiplasmodial Activity

The IC_50_ values of various combinations of *E. sigmoidea* and *T. macroptera* against chloroquine‐sensitive (3D7) and chloroquine‐resistant (Dd2) *P. falciparum* strains are listed here below in Table 3. All raw data, including IC_50_ calculations, are available in File S1. Combination 3 was better compared with the remaining two combinations based on IC_50_ values of 4.65 ± 0.09 and 4.67 ± 0.24 against chloroquine‐sensitive (3D7) and chloroquine‐resistant (Dd2) *P. falciparum* strains, respectively. Combinations of plant extracts were tested at concentrations expressed in micrograms per milliliter, whereas the positive control drugs, artemisinin and chloroquine, were tested at concentrations expressed in micromolar. Results are reported in accordance with standard practice for these assays. There was no significant difference (*p* > 0.05) among the IC_50_ of the various combinations.

### 3.3. Cytotoxicity Test

#### 3.3.1. Cytotoxicity on Vero Cells

The cytotoxicity results were shown in Table 4 below. It appears from this table that our combinations had no significant cytotoxic activity on Vero cells and that the combination (C_3_) had a IC_50_ of 217.05 ± 0.21 *μ*g/mL. A selectivity index greater than 10 indicates good selectivity toward the target with limited cytotoxicity to normal cells. Results are reported in accordance with standard practice for these assays.

#### 3.3.2. Hemolysis Test

**Figure 3:**
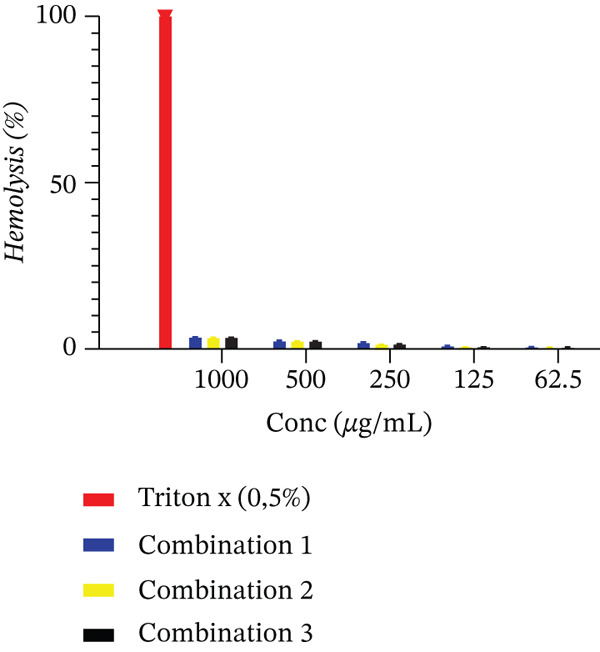
Hemolytic effect of combination of extracts from *E. sigmoidea* and *T. macroptera*.

Hemolysis percentages are used to express the hemolytic activity of the plant extract mixtures (Figure 3). When human erythrocytes were tested, various combinations showed very weak hemolytic activity.

### 3.4. Antioxidant Activities

Antioxidant activity of *E. sigmoidea* and *T. macroptera* combination extract is displayed in Table 5. The final extract combination concentration ranges from 12.5 to 200 *μ*g/mL (12.5, 25, 50, 100, and 200 *μ*g/mL). The combination blocked the DPPH (1, 1‐diphenyl‐2‐picrylhydrazyl) free radical from precipitating. IC_50_ value for the combination was 510.3 *μ*g/mL, whereas ascorbic acid was 4.718 *μ*g/mL. Sustainable reduction action was carried out by the combination. Vitamin C and combination IC_50_ values were undetermined and 159.6 *μ*g/mL, respectively. NO production was not substantially inhibited by the combination extract of *T. macroptera* and *E. sigmoidea*, and the activity was not concentration‐dependent. This indicates a pro‐oxidant effect against NO. For vitamin C and the combination, IC_50_ values were 54.22 *μ*g/mL and undetermined, respectively. Results are reported in accordance with standard practice for these assays. All raw data, including IC_50_ calculations for the antioxidant test, are available in File S2.

### 3.5. In Silico Approach and Molecular Docking Analysis

Glide module was employed to carry out molecular docking between the target protein and the ligands. Figure 4 illustrates the results. The interaction between the top five ligands and the target protein′s amino acids provided a high docking score. The docking scores for the top five ligands in relation to each other are listed in Tables 6 and 7 [35, 36].

Figure 43D and 2D interactions between *P. falciparum* dihydrofolate reductase‐thymidylate synthase (*Pf*‐DHFR‐TS) and ligands of *T. macroptera* and *E. sigmoidea* stem bark. (A) Sigmoidin A, (B) 3‐(2,4‐dihydroxyphenyl)‐7‐hydroxy‐6,8‐bis(3‐methylbut‐2‐enyl)‐2,3‐dihydrochromen‐4‐one, (C) Abyssinone V, (D) Flavogallonic acid dilactone, (E) (3R,5R)‐3,4,5‐tris[[(E)‐3‐(3,4‐dihydroxyphenyl)prop‐2‐enoyl]oxy]‐1‐hydroxy‐cyclohexane carboxylic acid, (F) chloroquin, (G) pyrimethamine.

**Figure:**
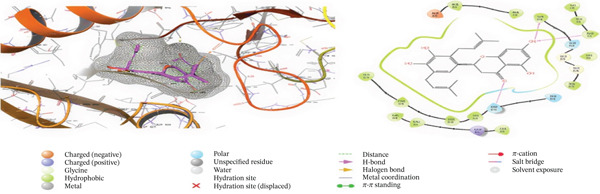
(A)

**Figure:**
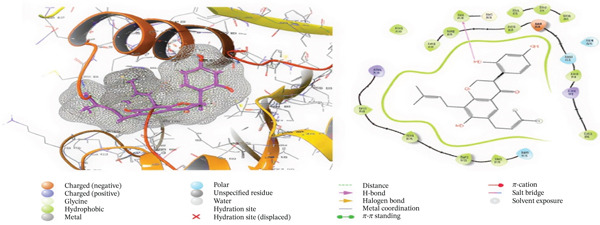
(B)

**Figure:**
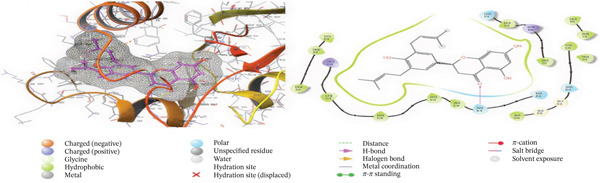
(C)

**Figure:**
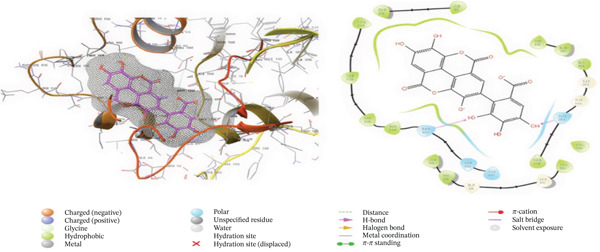
(D)

**Figure:**
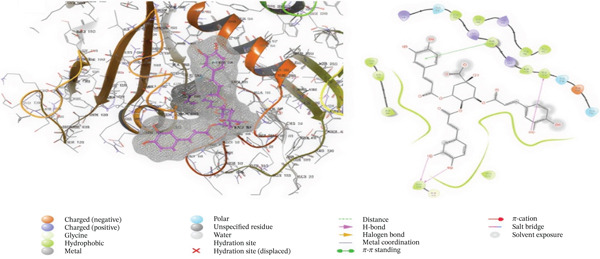
(E)

**Figure:**
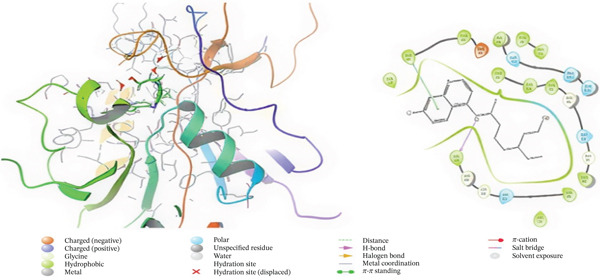
(F)

**Figure:**
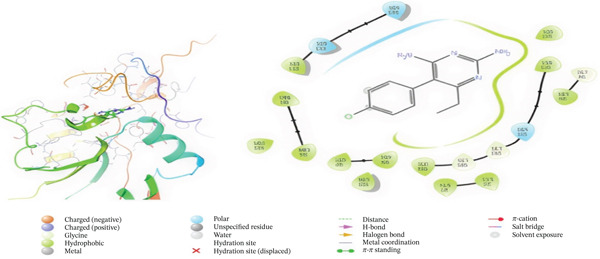
(G)

**Table 6:** *Pf*‐DHFR inhibitor binding affinity of compounds from *T. macroptera* and *E. sigmoidea* stem bark.

No.	Name of Compound	Scores		Interactions			Amino acids
		XPG Score/docking score	Glide Emodel	H‐bonds	*π*–*π*bonds	Salt bridges	
1	Sigmoidin A (CID: 73204)	−8.474	−73.282	3 (three) OH− Tyr170, Ala16‐OH, Ser111‐O	—	—	Tyr170, Ala16, Gly166, Ile164, Ser108, Ser111, Phe116, Leu119, Asp54, Met55, Phe58
2	Bidwillon A (CID: 10001497)	−8.029	−75.435	1 (one) OH‐ILE164	—	—	Cys59, Phe58, Ile164, Met55, Asp54, Asn51, Cys50, Lys49, Leu46, Ser111, Ile112, 3Pro113, Phe116, Phe116, Leu119, Arg122
3	Abyssinone V (CID: 442153)	−7.6	−64.158	1 (one) Ser111‐O	—	—	Asn51, Cys50, Lys49, Leu46, Ser108, Ser111, Ile112, Pro113, Phe116, Leu119, Arg122
4	Flavogallonic acid (CID: 71308199)	−7.579	−82.967	2 (two) Ser111‐OH, Ser111‐OH	—	—	Phe58, Met55, Leu119, Phe116, Pro113, Ile112, Ser111, Ser108, Thr107, Tyr170, Ser167, Gly166, Gly165, Ile164, Ala16, Cys15
5	3,4,5‐Tri‐O‐caffeoylquinic acid (CID: 626340)	−7.425	−87.563	3 (three) OH‐Ile112, OH‐Ile164, OH‐Ile164	Phe116	—	Ile164, Gly165, Met104, Ser108, Glu110, Ser111, Ile112, Pro113, Lys114, Phe116, Lys117, Leu119, Ser120, Arg122, Cys59, Phe58, Met55
6	Chloroquine (CID: 91971927)	−7.582	−67.194	N‐H, ILE 164			PHE 58
7	Pyrimethamine (CID: 4993)	−5.844	−44.320	2 (two) NH2‐Asn 108, NH2‐ Ala 16 NH2‐			Phe 52, Ile 112, Leu 119, Ala 16 Cys 15, Asp 54

**Table 7:** *Pf*‐DHFR inhibitor binding affinity of compounds from *T. macroptera* and *E. sigmoidea* stem bark using mutant (*Pf*‐DHFR‐TS) 8JFC.

No.	Name of compound	Scores		Interactions			Amino acids
		XPG score/docking score	Glide Emodel	H‐bonds	*π*–*π*bonds	Salt bridges	
1	Sigmoidin A (CID: 73204)	−8.215	−72.324	2 (two) OH− ASP 54 LEU 164	—	—	Leu 164, Asp 54, Met 55, Phe 58, Ala 16, Cys 15, Ile 14, Tyr 170, Asn 108, Ser 111, Pro 113
2	3,4,5‐tri‐o‐caffeoylquinic acid (CID: 6440783)	−8.177	−101.167	2 (two) OH− Leu 46 Pro 47	—	—	Val 195, Phe58, Ile164, Met55, Asp54, Asn108, Cys50, Lys49, Leu164, Ser111, Ile112, 3Pro113, Phe116, Phe116, Leu119, Arg122
3	Flavogallonic acid dilactone (CID: 71308199)	−7.796	−75.545	3(three) OH Asp 54 Gly 44 Ser 167	—	—	Val 45, Leu 46, Lys49, Leu46, Ser108, Ser111, Ile112, Pro113, Phe116, Leu119, Arg122
4	4‐Methylabyssinone V (CID: 6548074)	−7.616	−68.667	2 (two) Leu 164‐OH Asp 54‐OH	—	—	Phe116, Pro 113, Ile 112, Phe116, Pro113, Ile112, Ser111, Ser108, Thr107, Tyr170, Ser167, Gly166, Gly165, Ile164, Ala16, Cys15
5	Abyssinone V (CID: 442153)	−7.483	−66.806	2 (two) OH− Leu 154 OH− Asp 54 OH−		—	Ser 111, Asn 108, Met104, Ser108, Glu110, Ser111, Ile112, Pro113, Lys114, Phe116, Lys117, Leu119, Ser120, Arg122, Cys59, Phe58, Met55
6	Pyrimethamine (CID: 4993)	−5.908	−41.339	2 (two) NH2‐Asn 108 NH2‐Ala 16 NH2‐			Phe 52, Ile 112, Leu 119, Ala 16, Cys 15, Asp 54

The primary interactions between ligands and enzyme are H‐bonds. These H‐bonds are associated with the donation and acceptance of hydrogen between the ligands and the amino acids of the enzyme, which are Tyrosine 170, Alanine 16, Serine 111, Isoleucine 112, and Isoleucine164 Figure 5. These findings are consistent with previous research by Kumar et al. [37], Fogel et al. [38], Bhat et al. [39], and Saha et al. [40].

Figure 5(a) A Ramachandran plot of the *P. falciparum* dihydrofolate reductase‐thymidylate synthase (*Pf*‐DHFR‐TS) (PDB ID: 8JFC), and (b) an optimized version of the mutant (*Pf*‐DHFR‐TS).

**Figure:**
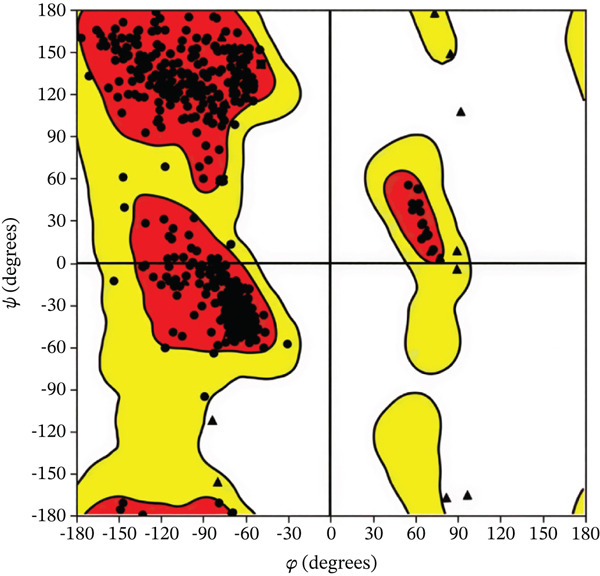
(a)

**Figure:**
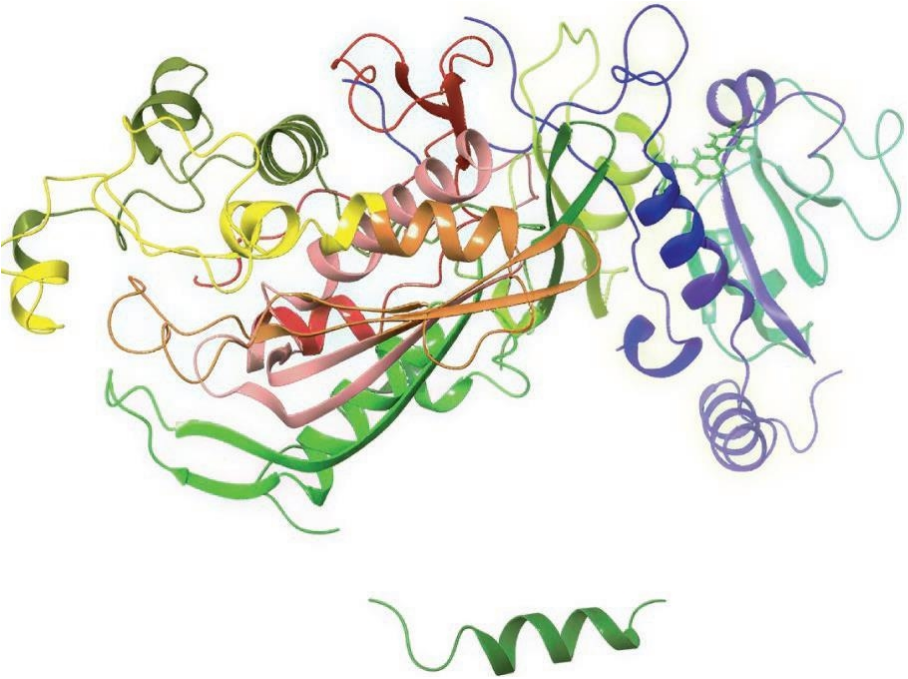
(b)

Figure 6 presents the 3D and 2D interactions between *Pf*‐DHFR‐TS and ligands of *T. macroptera* and *E. sigmoidea* stem bark using mutant (*Pf*‐DHFR‐TS) 8JFC. Two boxes of dimensions 15 x 15 x 15 and 20 x 20 x 20 (X:2.95, Y:29.15, Z:5.94) are constructed in order to find the grid center.

Figure 63D and 2D interactions between *P. falciparum* dihydrofolate reductase‐thymidylate synthase (*Pf*‐DHFR‐TS) and ligands of *T. macroptera* and *E. sigmoidea* stem bark. (a) Sigmoidin A, (b) 3,4,5‐tri‐o‐caffeoylquinic acid (c) flavogallonic acid dilactone, (d) 4‐Methylabyssinone V, (e) Abyssinone V, and (f) pyrimethamine.

**Figure:**
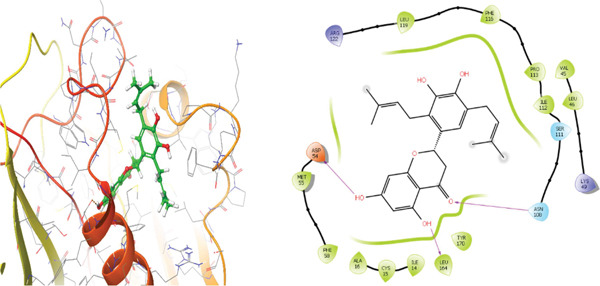
(a)

**Figure:**
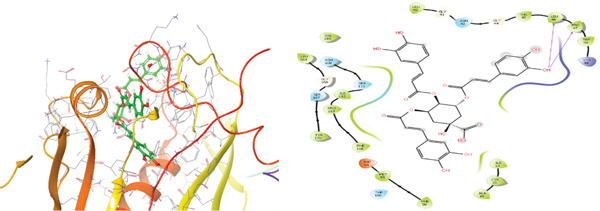
(b)

**Figure:**
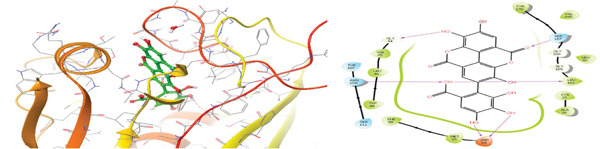
(c)

**Figure:**
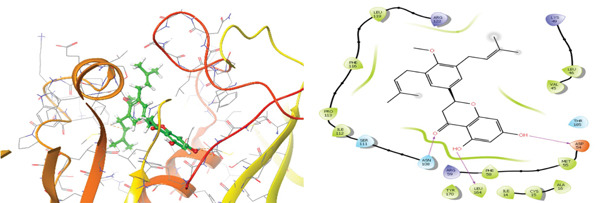
(d)

**Figure:**
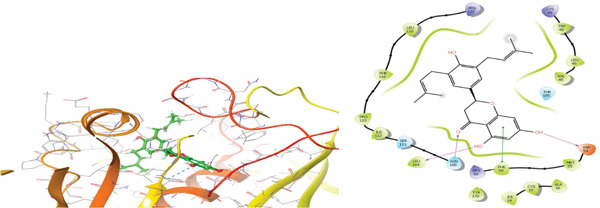
(e)

**Figure:**
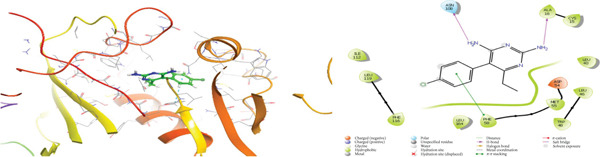
(f)

### 3.6. ADME Evaluation

To determine whether the identified compounds exhibit drug‐like behavior, the ADME properties were predicted in silico as it can be seen on Table 8. Several compounds exhibit a favorable ADME profile and do not violate any Lipinski rules. In addition, they do not possess any mutagenic or carcinogenic qualities. Based on these results, Compounds 1–3 have the potential to be developed into drugs.

**Table 8:** ADME scores for *T. macroptera* and *E. sigmoidea* stem bark compounds compared with *Pf*‐DHFR inhibitors.

No.	Compounds	Solute molecular weight^a^	QPlogHERG^b^	QPPCaco^c^ (nm/s)	QPPMDCK^d^ (nm/s)	Rule of five^e^	Rule of three^f^
1	Sigmoidin A (CID: 73204)	424.493	−4.842	91.197	37.172	0	1
2	Bidwillon A (CID: 10001497)	408.493	−5.389	417.925	192.665	0	2
3	Abyssinone V (CID: 442153)	408.493	−5.978	361.414	164.666	1	2
4	Flavogallonic acid dilactone (CID: 71308199)	454.303	−3.087	0.093	0.028	2	1
5	3,4,5‐Tri‐O‐caffeoylquinic acid (CID: 626340)	678.602	−6.6	0.04	0.011	3	3

^a^130–725.

^b^(concern below −5).

^c^ *a* < 25 is poor and *a* > 500 is great.

^d^ *a* < 25 is poor and *a* > 500 is great.

^e^Maximum is 4.

^f^Maximum is 3.

## 4. Discussion

This study is aimed at determining the in vitro antimalarial activity of a coextract of *T*. *macroptera* and *E. sigmoidea* stem barks against selected *Plasmodium* strains. The study was conducted to evaluate whether the combination of the two extracts exhibits greater activity compared with the individual effects of each extract. The efficacy of the mixture in inhibiting parasite proliferation was assessed, providing valuable information on its potential as a treatment and supporting the traditional use of these herbs in malaria management.

Three combinations of extracts were shown to inhibit *Plasmodium* strains by Singh et al.′s classification [41]. The IC_50_ values of Combination 3 against chloroquine‐resistant strains (Pf Dd2) and chloroquine‐sensitive strains (Pf 3D7) were lower (4.64 ± 0.09 *μ*g/mL and 4.67 ± 0.24 *μ*g/mL). A very active extract is one whose IC_50_ is below 5 *μ*g/mL, an active extract one that ranges between 5 and 50 *μ*g/mL, a moderately active extract one that ranges between 50 and 100 *μ*g/mL, and an inactive extract one that is above 100 *μ*g/mL, according to Kumar et al. We can therefore conclude that our extract works against the resistant and susceptible strains of chloroquine [37].

In this study, the CC_50_ value of the plant extract mixture was found to be 217.05 ± 0.21 *μ*g/mL. For the interpretation of cytotoxicity, it is generally accepted and was applied in this case that a CC_50_ below 1.0 *μ*g/mL indicates high cytotoxicity, 1.0–10.0 *μ*g/mL indicates moderate toxicity, 10.0–30.0 *μ*g/mL indicates mild toxicity, and values exceeding 30 *μ*g/mL are considered nontoxic [42]. According to these results, we conclude that the plant extracts did not exhibit cytotoxic effects. The significant antiplasmodial activity may be because the phytoconstituents of some plant extracts were targeting the membranes, cell vacuoles, or cytoplasm of the parasite [43]. Since the erythrocyte model gives a direct and general assessment of membrane toxicity, it has frequently been employed to evaluate cytotoxicity in erythrocytes [44]. This model has been utilized by a number of researchers in order to examine the interactions between drugs and plant extracts with membranes [45]. Upon hemolysis, the lipid bilayer membrane is broken and results in red blood cell rupture. The stability of the erythrocyte membrane was not affected whatsoever by the combination of plant extracts. Additionally, at 1000 *μ*g/mL, the blend was less toxic (less than 20%) to human erythrocytes than the positive control. The increased hemolytic activity levels in this study could be attributed to various factors like contamination, and the lytic activity of the extract or membrane instability. The study confirmed that the extract was not toxic and therefore could be utilized in treating malaria and other ailments.

The hydroxyl groups that have strong radical scavenging activity and the ability to donate electrons in order to nullify the free radicals might be the cause of the high antioxidant activity of the extract. Electron donation is required in a bid to curb the oxidative stress within the host [46]. This is evidenced by the DPPH experiment, where it is proven that the content of hydroxyl within the extract is accountable for its radical scavenging capacity [47]. By donation of hydrogen to suppress free radical chain reactions, antioxidants act as radical scavengers. Similar to ascorbic acid in the FRAP assay, the combination stem bark extract of *T*. *macroptera* and *E*. *sigmoidea* showed higher reducing power than controls with efficient reduction of Fe^3+^‐TPTZ to Fe^2+^‐TPTZ, according to experimental data. It reflects the potency of the extract as a redox‐active agent [48].

The strong antioxidant activity of the extract may be attributed to electron‐donating hydroxyl groups, which can effectively neutralize free radicals. Such electron donation is essential for reducing oxidative stress in the host. This effect is demonstrated by the DPPH assay, where the presence of hydroxyl groups in the extract plays a key role in its radical‐scavenging activity [49]. To prevent free radical chain reactions, antioxidants donate a hydrogen atom. Similar to ascorbic acid, the combined stem bark extract of *T*. *macroptera* and *E*. *sigmoidea* had a higher reducing ability than the control in the FRAP assay, effectively reducing Fe^3+^‐TPTZ to Fe^2+^‐TPTZ. These results confirm the extract’s high redox‐active activity [50].

NO is one example of a free radical derived from nitrogen that takes part in the generation of other reactive species. NO has the distinction of being an important regulator of vascular homeostasis, namely blood pressure, in addition to being a free radical. In this work, the ability of plant extracts to inhibit NO generation in vitro was investigated. The results showed that, contrary to vitamin C, the extracts seemed to augment the production of NO from sodium nitroprusside rather than inhibit it. This suggests that rather than inhibiting NO production, the combination of plant extracts can provoke it. However, since increasing NO bioavailability is thought to be therapeutically significant, this action may prove useful in the management of hypertension.

We have identified a number of compounds through liquid chromatography. We have listed the five best compounds that have the highest score in docking. Virtual screening revealed the putative target of plasmodia, partner protein interacting with Sigmoidin A. They found that the related flavanone Sigmoidin A purified from *Erythrina abyssinica* displayed in vitro antiplasmodial activities in the range of 5.9–13.6 *μ*M against *P. falciparum* Strains D6 and W2. This good molecular affinity that we noticed could be in agreement with this biological data. The constitution of Sigmoidin A, particularly its flavanone core having prenyl groups, provides good opportunities for hydrophobic and *π*–*π* interaction with the active site of the plasmodial target that justifies its antimalarial potential. Preliminary ADME profile indicates moderate solubility and low in silico toxicity prediction values, in line with natural flavanones [51].

In our simulations, Bidwillon A exhibited moderately strong binding. Although Bidwillon A is not well‐known for its antiplasmodial activity, it has demonstrated notable antibacterial efficacy in *E* *r* *y* *t* *h* *r* *i* *n* *a* × *b* *i* *d* *w* *i* *l* *l* *i* *i*, particularly with very low MICs against some oral bacteria, including *Fusobacterium*. Its capacity to bind to bacterial protein channels indicates some potential for binding to parasitic proteins as well. Our docking studies support this binding affinity, even in the absence of direct antiplasmodial evidence, and thus Bidwillon A may be considered for further in vitro investigation on *Plasmodium* [52].

Abyssinone A exhibited some of the highest docking scores alongside favorable binding geometry. It is one of the flavanones extracted from *E*. *abyssinica* with IC_50_s ranging from 4.9 to 13.6 *μ*M for the D6 and W2 strains. Thus, it has been proven to possess antiplasmodial activity. The expected binding affinity corroborates with the activity observed in vitro. Additionally, the presence of the prenylated flavanone core increases directed hydrophobic interactions [53].

Little research has been done on the direct antiplasmodial effects of flavogallonic acid, but polyphenols that contain gallic acid are known to exert antioxidant effects, in some cases even antimalarial effects, due to their modulation of plasmodial oxidative stress. Flavogallonic acid′s moderate affinity may be explained by its capability to form multiple hydrogen bonds with the target′s polar residues. Despite being less hydrophobic, its hydroxyl group′s lipid irrationality translates to some degree of complementarity to hydrogen bond dictated enzyme active sites. Although the ADME profile indicates poor oral absorption, the compound′s hydrophilic affinity proposes reasonable moderate potential to be investigated with appropriate intervention [54].

This particular acylated quinic acid derivative exhibited the most favorable binding affinity in our simulations. Although caffeoylquinic acid esters have not been directly tested against *Plasmodium*, it is known that chlorogenates frequently possess antioxidant properties and the ability to inhibit certain microbial enzymes. The presence of many hydroxyl groups and the multilayered acylation of the compound translates to a greater surface area which leads to strong binding interactions (hydrogen bonds and *π* interactions) and thus, an excellent docking score. The large size of the compound ensures that it can effectively block the targeted enzyme active site [55].

## 5. Conclusion

In the Western Region of Cameroon, traditional healers employ a blend of *T*. *macroptera* and *E*. *sigmoidea* to treat malaria. The ethanol stem bark extract of these plants has shown low cytotoxicity, both antioxidant activity, and activity against two strains of *P*. *falciparum*: one chloroquine‐sensitive (3D7) and the other chloroquine‐resistant (Dd2). Also, to validate the use of these plants, in vivo antimalarial and toxicity studies should be performed to confirm the usage of *T. macroptera* together with *E. sigmoidea* to treat malaria because the activities of the two plants separately have already been confirmed. It is possible to conclude that some of the primary compounds of *T. macroptera* and *E. sigmoidea* are deserving of further study into their potential therapeutic uses based on docking simulations and the ADME characteristics of the five ligands with the highest interaction with the target enzyme.

## Author Contributions

The test was planned and the concept was conceived by T.N.J.S., Y.C., N.A.C.N., V.K.P., and H.H. Experiments were conducted by Y.C., N.A.C.N., T.N.J.S., G.N.G‐A., N.N.A.S., and M.A.A. Analysis and evaluation of data were done by T.N.J.S. and Y.C. The manuscript was prepared by T.N.J.S., N.A.C.N., Y.C., and V.K.P. All authors reviewed the final draft.

## Funding

This study was supported by the Scientific Research Key Project of the Jiangxi Education Department (GJJ211501).

## Disclosure

All authors approved the final draft.

## Conflicts of Interest

The authors declare no conflicts of interests.

## Supporting Information

Additional supporting information can be found online in the Supporting Information section.

## Supporting information


**Supporting Information 1** File S1: Raw experimental data used for the determination of IC_50_ values in the cytotoxicity and antiplasmodial assays, including the datasets and calculations employed for IC_50_ determination.

**Supporting Information 2** File S2: Raw experimental data and calculations used for the determination of IC_50_ values in the antioxidant assays.

## Data Availability

Upon reasonable request, the corresponding authors will provide the data supporting the conclusions in the study.
